# Trusted messengers in everyday spaces: leveraging community expertise to support early language and literacy

**DOI:** 10.3389/fpubh.2026.1980834

**Published:** 2026-10-22

**Authors:** Susan B. Neuman, Lauren Krieger

**Affiliations:** Department of Teaching and Learning, New York University, New York, NY, United States

**Keywords:** community engagement, early childhood development, early literacy, health equity, informal learning environment, social determinants of health, trusted messengers

## Abstract

**Introduction:**

Informal community spaces may offer an underused opportunity to support early language and literacy, particularly when families encounter trusted individuals who understand their communities. This study examined how trusted messengers foster early language and literacy in everyday community settings and considered the relevance of this approach for public health efforts to reduce disparities in early childhood opportunity.

**Methods:**

We drew on three design studies conducted in laundromats across New York, Chicago, and Philadelphia. Environmental redesigns created opportunities for literacy-related play, while public librarians and Reading Captains—community volunteers with cultural and linguistic ties to families—served as trusted messengers. Qualitative observations, field notes, and caregiver reports were used to examine how these messengers engaged children and caregivers within the redesigned spaces.

**Results:**

Environmental redesigns increased children’s literacy-related play, but the presence of trusted messengers generated more sustained engagement. Librarians provided structured, professionally guided activities resembling story hours, extending children’s exposure to literacy-rich routines. Reading Captains built relational trust through everyday conversation, bilingual exchanges, and culturally familiar practices, fostering caregiver participation and affirming families’ existing practices. Both approaches deepened engagement, though in distinct ways.

**Discussion:**

The findings suggest that physical environments alone may not be sufficient to sustain engagement. Trusted messengers can help transform everyday community spaces into settings that support children’s early language and literacy while strengthening connections with caregivers. Because this approach builds on existing community spaces, relationships, and expertise, trusted messengers may represent a scalable component of place-based public health strategies designed to expand early developmental opportunity, community engagement, and equity.

## Introduction

Despite decades of investment, persistent disparities in early language and literacy development continue to mirror broader patterns of racial and economic inequality (1). Children in under-resourced communities often face limited access to high-quality early education, few opportunities for sustained, language-rich interactions, and significant barriers to books, materials, and enrichment activities (2, 3). These gaps emerge early and tend to widen over time, shaping school readiness, academic trajectories, and children’s developing identities as learners (4). From a public health perspective, these disparities matter beyond school readiness: education is a well-established social determinant of health, with pathways through health literacy, health behaviors, economic opportunity, and long-term well-being (5).

In response, the national conversation on early literacy has increasingly emphasized evidence-based instruction, early intervention, and equitable access to high-quality programs (6). Yet much of this discourse has remained centered on formal systems--schools, curricula, and teacher preparation. While these institutions are undeniably central to children’s language and literacy development, they are not the only spaces where learning unfolds, nor are teachers the only adults shaping literacy trajectories. Too often, we have overlooked the informal, everyday environments where young children spend over 80% of their time (7). For public health, these settings are especially important because they offer opportunities to reach families outside formal service systems and to build developmental supports into the places and routines of daily life.

This conventional focus has often led to undervaluing the powerful role of the local community and its members in supporting early literacy. In contrast, this study explores what becomes possible when we shift our gaze beyond formal systems and consider the informal, everyday spaces where children live, play, and grow. Central to this exploration is the role of the trusted messenger, local community members who have earned the confidence of families and serve as credible sources of support and information (8). Trusted-messenger approaches have also gained attention in public health as a strategy for reaching communities that may be poorly served by, or less trusting of, formal institutions (9). These individuals, who are often deeply embedded in the cultural and social fabric of children’s neighborhoods, can foster literacy-rich interactions that feel natural, relevant, and affirming.

In this research, we explore how an informal, community-based setting can support early language and literacy development by leveraging the relational strengths of trusted community members. We situate this work in an unconventional yet familiar space: the neighborhood laundromat. More than a place for routine tasks, the laundromat can function as an organizational broker--a community anchor that fosters social connection, circulates information, and links families to local resources (10, 11). Within this setting, we focus on ways in which trusted messengers engage children in playful, everyday interactions that blend talk, play, and literacy-related activity in a context of linguistic and cultural familiarity. Our goal was to examine how these relationships, grounded in trust and shared experience, might expand access to literacy opportunities and help reduce long-standing inequities in children’s learning environments. In public health terms, the model tests a place-based, community-engaged approach to strengthening a developmental resource before disparities become more entrenched.

This study is grounded in a socioecological perspective (12), which conceptualizes child development as occurring within a series of nested, interdependent systems, from immediate family to broader societal structures. From this perspective, children’s learning is shaped not only by what happens in schools and homes, but also by the larger ecological contexts in which families live, including community spaces, neighborhood institutions, and informal relationships. These mesosystems and exo-systems can either constrain or expand opportunities for learning, especially in under-resourced communities where structural inequities limit access to formal supports (13). This ecological framing is also consistent with public health approaches that locate developmental opportunity within social and community conditions rather than solely within individual children or families.

We draw further on sociocultural theory, particularly Vygotsky’s notion that learning is mediated through social interaction and cultural tools (14). Literacy is not simply a set of technical skills, but a culturally situated practice that develops through participation in meaningful activities with others (15, 16). These shared activities such as storytelling, playful talk, or book reading help children make sense of their world and lay the foundation for formal literacy. In line with this view, everyday community settings like laundromats can function as activity settings (17), where language development is supported through roles, scripts, and interactions embedded in familiar cultural routines.

A third strand of this framework draws from research on organizational and relational brokerage. Community institutions--such as barbershops, churches, or childcare centers--may serve as organizational brokers, connecting families to resources and information that support child development (18, 19). Within these spaces, relational brokers, individuals who are culturally and socially embedded in the community can build trust, share knowledge, and strengthen informal support networks. These trusted messengers operate not as formal experts, but as credible, empathetic guides who meet families where they are (8, 20). Previous studies, for example, have shown how nurses and librarians can act as trusted messengers (21, 22), conveying important information in contexts that build information and social support.

Finally, our work is informed by the principles of culturally sustaining pedagogy (23), which aim to support both access to dominant literacies and the preservation and development of children’s cultural and linguistic identities. In contrast to deficit-based models that assume communities lack capacity, this framework emphasizes the importance of affirming children’s home languages, cultural practices, and lived realities as assets in learning. Recent research on translanguaging (24, 25), conversational turn-taking (26), and language development in informal spaces (27, 28), for example, underscores the importance of responsive, culturally grounded exchanges in shaping literacy trajectories. By situating literacy engagement in community-driven spaces and relationships, our study aligns with a broader shift toward ecologically valid and culturally responsive approaches to early learning (29–31). This emphasis on cultural and linguistic fit parallels public health evidence that community-based, culturally concordant messengers can improve reach, trust, and engagement among populations that experience barriers to conventional services (32). Taken together, this theoretical lens allows us to explore not only what supports early literacy, but where, how, and with whom it can be meaningfully developed.

Our research is based on a series of design studies examining how a community-based space such as the laundromat can be leveraged to support early literacy. Design studies are research approaches that develop, test, and refine educational interventions in authentic contexts (33). Using a similar methodology across three investigations, we first conducted a trial in New York City. Following baseline observations, three laundromats were redesigned with books, toys, and materials to spark children’s reading and play, while three comparable sites remained unchanged. Using time-use diaries, research assistants recorded the frequency of literacy-related activities in two, 2-h time blocks for 8 weeks. The differences were striking: children in the redesigned laundromats engaged in significantly more literacy-related activities, demonstrating that modest design changes could alter children’s behavior in meaningful ways. In the next 8-week phase, public librarians joined these sites as trusted messengers. During their 2-h visits each week with children, they read stories and led literacy-related activities. With their involvement, children’s engagement was not only more frequent but also more sustained--play became richer, longer, and more explicitly focused on literacy.

A second study in Chicago extended this work to 20 laundromats across diverse neighborhoods. Once again, the combination of environmental redesign and librarian involvement significantly enhanced children’s literacy engagement, roughly 40–50% compared to design-only periods. Yet despite these benefits, the librarians’ position as outsiders often limited the extent to which parents and caregivers became actively involved. Therefore, in a third study, we addressed this limitation by introducing Reading Captains--community volunteers who shared the cultural and linguistic backgrounds of the families they served. As trusted insiders, Reading Captains fostered sustained, high-quality interactions that engaged not only children but also caregivers in meaningful literacy activities with increases closer to 60% and with richer caregiver participation. Their presence transformed the laundromat into a vibrant hub where families felt welcomed, affirmed, and connected. Figure 1 highlights these results in all three cities.

**Figure 1:**
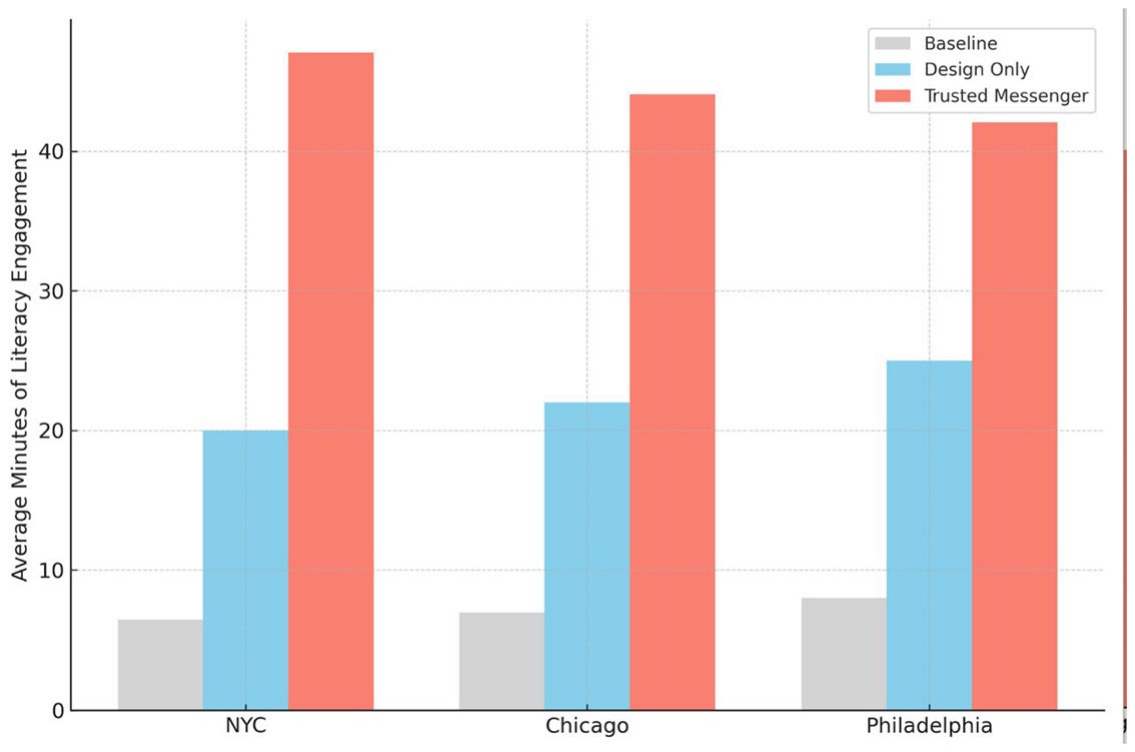
Impact of design changes versus trusted messengers across sites.

Together, these three studies demonstrated that while design changes can spark literacy activity, it is the trusted messenger—whether a credentialed professional or a community insider—who deepens and sustains engagement, raising important questions about the role of trust, cultural responsiveness, and the institutional settings that make such relationships possible.

These studies, highlighting the pivotal influence of the trusted messenger, prompted us to look more closely at how this role shapes engagement. In this analysis, we addressed the following questions:

1. What practices and qualities enable trusted messengers to build authentic, nonjudgmental relationships with families, and how do these differ across insider and outsider roles?
2. In what ways do trusted messengers incorporate culturally and linguistically responsive strategies in their interactions, and how do these practices shape families’ willingness to engage in literacy opportunities?
3. What lessons across these studies inform the scaling and sustainability of trusted messenger models, and how might they contribute to more equitable and culturally sustaining approaches to early language and literacy?

## Materials and methods

### Settings and participants

To address these questions, this study draws on a cross-study re-analysis of our three design-based investigations conducted in New York City, Chicago, and Philadelphia, each of which examined how laundromats could be leveraged as community-based spaces to support early literacy. Across the three studies, the research was conducted in 29 laundromats: six in New York City, 20 in Chicago, and three in Philadelphia. While the original studies reported on the effects of environmental redesign and the presence of trusted messengers, the present analysis brings these datasets together to address a set of questions about the comparative role of trusted messengers across contexts.

Our studies were conducted in laundromats located in communities identified in partnership with the LaundryCares Foundation, which worked with local laundromat owners and community partners to establish participating sites. Because the studies took place in naturally occurring community settings, families were not recruited as a fixed cohort; rather, observations captured children and caregivers who visited the laundromats during scheduled observation periods. The duration and number of observation periods varied according to the design and context of each study.

Across the three studies, about 20 individuals served as trusted messengers, most of whom were women. All participated voluntarily, motivated by an interest in extending literacy opportunities to families in the communities they served. In New York and Chicago, public librarians assumed this role; in Philadelphia, community volunteers served as Reading Captains.

Reading Captains were selected with attention to their familiarity with the community and to the racial, ethnic, cultural, and linguistic characteristics of the neighborhoods surrounding participating laundromats. They generally worked in pairs, with the same individuals often returning to a particular laundromat across multiple visits to provide continuity for children and their families. To prepare for their role, trusted messengers were required to participate in a brief training, typically a day and a half. In New York and Chicago, librarians were trained by the Director of Children’s Services; in Philadelphia, Reading Captains completed online preparation provided by Too Small to Fail, an initiative of the Clinton Foundation, which included trauma-informed approaches to engaging children and families. Training was designed to provide a common foundation for the work while allowing trusted messengers to draw on their own professional, cultural, and community knowledge in responding to families.

Prior to data collection, IRB approval was received for each study through our institution (New York University). Given that the review provided assurances that privacy and confidentiality protections were adequate and that the project presented no risk to participants, we were not required to collect informed consent or debrief participants.

Across these studies, data sources included systematic observations of children’s literacy-related activities using time-use diaries; low-inference field notes documenting child and caregiver interactions; parent and caregiver brief interviews of the literacy-related play spaces and trusted messengers; and researcher memos and photographs capturing the organization of space. The studies varied in duration and design, but each included repeated observations over time, beginning with observations of naturally occurring activity and examining subsequent changes associated with literacy-related environmental supports and the presence of trusted messengers. Since our goal was to examine the role of trusted messengers in depth, we focus here on our qualitative observations. Detailed descriptions of site selection, research design, observation schedules, measures, and quantitative analyses are reported in our earlier studies (10, 21, 34).

### Data analysis

All qualitative data were imported into MAXQDA, a qualitative software program, for analysis. To ensure consistency across sites, we developed a shared codebook that drew both from our prior work on literacy engagement (10) and inductive codes that emerged during familiarization with the data. Following Braun and Clarke’s framework for thematic analysis (35, 36), we engaged in several phases of coding. We read through all our notes to generate initial codes, highlighting phrases or actions that seemed to exemplify those initial impressions. We used an iterative process, working through each dataset separately (NYC, Chicago, Philadelphia), then synthesizing our themes to identify commonalities and contrasts in the role of trusted messengers across contexts.

We established inter-rater reliability by having two researchers independently code a subset of field notes and transcripts. Agreement exceeded 85%; discrepancies were resolved through discussion, leading to a further refinement of the shared themes. This process not only improved consistency but also ensured that themes were not idiosyncratic to a single analyst or a particular dataset. Following our analysis, we shared our themes with Reading Captains, librarians and community stakeholders, inviting feedback on whether the findings reflected their experiences. This process of member checking allowed us to verify the accuracy of our interpretations, ensure that the themes resonated with participants’ lived experiences, and refine our analysis based on their feedback. Incorporating their perspectives strengthened the credibility and trustworthiness of the findings.

## Results

In the following results, we highlight how trusted messengers in our studies, both credentialed professionals and community insiders, shaped children’s literacy engagement and family participation in laundromats. In what follows, we describe the distinctive practices and qualities that characterized each group of messengers, as well as the ways laundromats functioned as organizational brokers linking families to literacy opportunities.

### Practices and qualities of trusted messengers

Across all sites, the presence of trusted messengers consistently deepened and sustained children’s literacy engagement. In New York, for example, children remained in literacy play areas for an average of 47 min—a substantial amount of engaged time compared to the 6.5 min observed when they played alone. Similar patterns were documented in Chicago and Philadelphia, with children spending nearly twice as much time engaged in literacy activities when trusted messengers were present.

The structure and style of these sessions, however, varied markedly between librarians and Reading Captains. In New York and Chicago, public librarians drew on their professional training to create well-organized sessions that brought a story-hour atmosphere into the laundromat. Sessions often began with songs, moved into interactive read-alouds, and incorporated prompts and questions to scaffold children’s talk. While attentive to the informal context, librarians emphasized teaching for literacy engagement (37)—helping children enjoy and interact with texts while connecting their experiences to the local environment. Because children could leave at any time, librarians adapted quickly, shifting to hands-on activities, applying stories to the immediate setting, or dropping planned activities to follow a child’s lead. Even with such flexibility, their sessions remained clearly planned and goal-directed. They were language-rich, with activities designed to support oral language development, phonological awareness through rhymes and songs, and even math concepts such as shapes, numbers, and patterns found in the laundromat. In short, librarians did what they do best: immersing children in a diverse range of high-quality literacy experiences.

Parents, however, tended to remain at the periphery during these sessions. Only 17% of parent–child interactions involved direct participation. Most often, parents praised their child’s work, reviewed artwork, or snapped photos with evident pride while letting the librarian lead the activity. As one note described, parents “usually let the librarian do the activity, participating along only on rare occasions.”

By contrast, the Reading Captains in Philadelphia assumed a broader, more relational role. They engaged not only in reading but also in informal talk and practical support that connected families to everyday needs. Closely tied to the neighborhood, Captains often began conversations about schools, favorite teachers, or shared community experiences. They celebrated milestones, giving books to children for birthdays, and responded to questions from parents. On one occasion, a mother asked, “How do I know when to sign him up for kindergarten?” The Captain offered specific guidance about deadlines and forms. In another, a grandmother joined in a story session, reminiscing about “the way we used to tell stories back home,” weaving her memories into the moment. These encounters were often impromptu and responsive, designed less around a structured plan than around opportunities as they arose. In doing so, Captains created a sense of belonging and nonjudgmental support, where families felt both seen and affirmed.

This distinction between these roles of trusted messengers should not be understood as a preference for one approach over another. Children clearly need foundational skills and knowledge to become proficient readers, but they also benefit from developing broader personal and social resources. Prior studies confirm that social–emotional competencies are closely tied to language and literacy outcomes, both concurrently and longitudinally (38). In their interactions, Reading Captains conveyed implicit understanding that children’s everyday experiences were heard. For instance, when a child shared a personal story about being bullied in school, a Captain responded with warmth and empathy, suggesting several ways the problem might be resolved. Such validation reflected a more holistic view of what young children need to thrive. In everyday spaces like laundromats, caring adults inside and outside the family can provide the security, recognition, and sense of identity that nurture confident, capable learners.

### Cultural and linguistic responsiveness in action

The second question addressed the ways in which trusted messengers interacted with community members. Throughout the data, it was evident that trusted messengers engaged in practices that affirmed and drew upon the everyday languages, traditions, and experiences of families, rather than positioning them as deficits or obstacles. In community spaces like laundromats, responsiveness involved more than simply adapting a literacy activity; rather, it recognized families’ ways of speaking, remembering, and celebrating as integral to children’s learning.

Reading Captains embodied this approach through relational, conversational practices that blurred the line between literacy and everyday life. They often shifted easily between English and Spanish, signaling that bilingual expression was not only accepted but valued. One Captain described a moment when a child began moving between languages: “He switched back and forth, and I just nodded and said, ‘Sí, I understand, you can say it both ways.’ He smiled so big — it was like he felt seen.” Such exchanges reminded children that their linguistic practices belonged in this space.

Captains often drew on everyday routines to connect literacy with family life. One Captain noticed a mother folding laundry and offered, “Would you like me to read with him while you finish folding?” The mother smiled and said, “That would be amazing—he loves books, but I can never get him to sit still.” What followed was a lively read-aloud that not only held the child’s attention but soon drew the mother in as well. On another occasion, a Captain paused during a story about gardening to ask a child, “Do you help water the plants at home?” The child eagerly replied, “Yes, with my grandma!”—prompting the Captain to weave the child’s own family routines into the story. Captains also celebrated birthdays by giving children books, turning literacy into part of family ritual and joy. These gestures positioned reading as something rooted in belonging and affection, not only instruction.

Librarians, on the other hand while working from a different stance, also demonstrated responsiveness by adapting their professional practices to an informal, unpredictable setting. Their sessions often began with songs and rhymes familiar across communities, creating an inviting rhythm that quickly drew children in. One librarian explained, “We never knew how long we had — sometimes only a few minutes before the washer buzzed — so I would start with a song that every child could join, no matter their age or language.” They frequently paused read-alouds to ask children to connect text to their surroundings: “Can you find this shape on the washer?” or “Who here has ridden a bus like this one?” When attention flagged, they set aside books for puppets, counting socks, or naming colors in the room. These adaptations acknowledged children’s interests while still keeping the session literacy-rich and purposeful.

Taken together, both types of messengers enacted cultural and linguistic responsiveness, but in distinct ways (see Table 1). Reading Captains emphasized *relational affirmation*—meeting families where they were, celebrating their stories, and weaving literacy into the fabric of everyday life. Librarians emphasized *adaptive expertise*—bringing structured, evidence-based routines into the laundromat, but adjusting them fluidly to context. Both approaches created authentic, welcoming spaces in which children’s languages, cultures, and identities were affirmed as central to learning. Together, these findings suggest that both forms of responsiveness—professional structure and relational affirmation—are complementary, each offering families meaningful but distinct pathways into literacy.

**Table 1:** Practices and qualities of trusted messengers in laundromats.

Trusted messenger	Specific practices	Qualities demonstrated	Examples from field notes
Public Librarians (NYC/Chicago)	Organized sessions resembling story hour (songs, fingerplays, interactive read-alouds) Asked questions to connect stories to children’s lives (e.g., ‘Have you ever been on an airplane?’) Improvised when attention waned (puppets, shifting to hands-on activity) Drew on laundromat environment (counting socks, naming shapes/numbers on machines)	Professional expertise Structured and goal-directed Flexible within informal setting	Sessions started with fingerplays followed by book reading, active games, pretend play, and writing activities
Reading Captains (Philadelphia)	Shifted fluidly between English and Spanish Used familiar cultural references (foods, music, neighborhood talk) Gave books as birthday gifts Invited families to share traditions and memories	Insider status, cultural familiarity Relational and approachable Responsive to family needs	RC1: Do you want to read in English or in Espanol? Child #1: I do not know. RC1: Why do not we read a little in English, and a little in Espanol? The girl nods. RC1 selects a picture book from a pile nearby.

### Scaling and sustaining trusted messenger models

The third research question asks what lessons can be drawn from these studies for scaling and sustaining trusted messenger models. What emerges most clearly is that these efforts are not simply about placing books or activities into new locations but about cultivating relationships that are durable, meaningful, and grounded in community life.

#### Scaling

Across cities, the adaptability of trusted messengers suggests the potential for reaching families wherever they naturally gather. Laundromats proved powerful because they are routine, familiar, and accessible; but the principle can extend to barbershops, clinics, corner stores, or playgrounds. What matters is not the site itself but the way trusted messengers transform it--turning ordinary waiting spaces into hubs of learning and connection. The studies also show that there is no single formula. Librarians offered structured, well-crafted literacy experiences that made story hour portable, while Reading Captains brought neighborhood knowledge and cultural familiarity that invited families to see themselves reflected. Scaling, then, means allowing communities to shape the model to their own contours rather than imposing a uniform design.

#### Sustainability

These efforts endure when they are woven into the fabric of community institutions. Librarians could sustain their role when libraries recognized outreach as part of their mission. Reading Captains thrived when neighborhoods embraced them as insiders, neighbors, and advocates. Sustaining trusted messengers requires partnerships that cross organizational boundaries--libraries, schools, community groups, and funders working together but it also requires something less tangible: a sense of ownership. When families see trusted messengers as “ours,” not “theirs,” programs cease to feel temporary and become part of the rhythm of everyday life.

#### Equity and culturally sustaining approaches

Above all, these studies show that trusted messengers can help reimagine what equity in early literacy might look like. Equity is not only about providing equal access to books and instruction; it is about affirming families’ voices, languages, and ways of telling stories. In laundromats, parents and grandparents shared memories of how stories were told “back home,” and Captains affirmed children’s playful expressions as worthy contributions. Librarians, too, found ways to link books to children’s lives, turning machines into counting tools and rhymes into playful songs. In these small acts, families experienced literacy not as a measure of what they lacked but as an extension of who they already were. Scaling trusted messenger models, then, is not about replicating programs at larger scale, but about nurturing ecosystems of care where families feel recognized, respected, and welcomed into the circle of literacy.

Consequently, questions of sustainability and scale often begin with what a new program will cost. Our findings suggest a different starting point: what resources are already present in communities, but have not yet been well-connected. These laundromat projects required commitment more than capital. Librarians saw community outreach as part of their professional mission and brought their expertise beyond the walls of the library. Reading Captains gave their time because they cared about children, literacy, and their neighborhoods. A local foundation supplied books and materials, including resources that reflected the languages of participating families. What made the model possible, then, was not a costly new infrastructure, but the intentional connection of assets that already existed--people willing to serve, institutions willing to reach outward, community spaces willing to open their doors, and philanthropic partners willing to provide materials. Sustaining that network still requires coordination, continued investment, and an adequate supply of culturally and linguistically relevant books; it cannot depend on goodwill alone. And that may be the promise for scale. Rather than asking communities to build something entirely new, this approach asks how we might better connect and support what is already there.

## Discussion

This study explored how neighborhood laundromats--ordinary, everyday spaces--can be reimagined as contexts for early literacy engagement, especially in communities historically underserved by formal educational systems. Across three cities, modest design changes created more opportunities for literacy play, but it was the presence of trusted messengers that transformed these spaces into vibrant hubs of learning and connection.

Our findings show that the practices and qualities of trusted messengers mattered deeply. Public librarians, drawing on their professional expertise, created structured, language-rich sessions that mirrored the best of story hour, but adapted nimbly to the unpredictable rhythms of the laundromat. Reading Captains, by contrast, relied on their insider status to weave literacy into everyday conversations, affirming children’s cultural practices and inviting families into the circle of participation. Both approaches were strong, though their emphases differed: librarians offered professional scaffolding and exposure to rich literacy routines, while Captains cultivated relational trust and cultural resonance. Together, they highlight the value of multiple pathways for building authentic, nonjudgmental relationships with families.

Equally important, these studies illustrate how cultural and linguistic responsiveness shaped families’ willingness to engage. When Captains responded to a child’s code-switching with “Sí, I understand, you can say it both ways,” or when librarians turned a washing machine into a tool for counting, families experienced literacy as something that connected to their own lives and languages. Such practices reinforced that children’s ways of speaking, playing, and remembering were not only accepted but celebrated. In this way, trusted messengers helped shift the narrative away from deficit-based views of what families lack, toward affirming the strengths they already bring (see 30, Principles 2, 3, and 4).

Looking across sites, these findings suggest that the promise of trusted messengers lies not in replicating a single model, but in adapting it to the places, relationships, and resources of local communities. For public health, this is a community-engagement lesson as much as an educational one: interventions may be more reachable and acceptable when they are embedded in trusted places, delivered through relationships, and shaped with rather than simply delivered to communities.

In sum, this study contributes to a growing body of research demonstrating the potential of trusted messengers to transform informal community settings into spaces of literacy, affirmation, and belonging. Whether through the structured expertise of librarians or the relational warmth of Reading Captains, these adults helped families imagine literacy not as an obligation or a gap to be filled, but as part of the everyday fabric of community life. Because early developmental opportunity and educational attainment are linked to health across the life course, strengthening such opportunities in under-resourced communities may also be understood as an upstream, equity-oriented public health strategy (5).

### Limitations

While these studies provide important insights, several limitations must be acknowledged. First, the interventions were relatively small in scale and situated in particular neighborhood contexts. Laundromats in New York, Chicago, and Philadelphia each reflected unique community dynamics, and results may not translate in exactly the same way to other informal settings. Second, our observations were conducted over a limited period, which allowed us to capture rich snapshots of practice but not the longer-term durability of change. We cannot know from these data whether children’s and families’ behaviors would persist beyond the study period, or whether trusted messenger roles might evolve over time. Third, the study relied primarily on qualitative observations, interviews, and field notes (39, 40). While these methods allowed us to capture the texture of interaction, they are subject to interpretation and do not permit causal claims. We also did not measure health outcomes; the public health implications advanced here rest on the established role of education and early developmental opportunity as upstream determinants and should be tested directly in future research. Finally, the trusted messengers themselves were deeply embedded in their communities—a strength of the model, but also a reminder that replicability will depend on finding or cultivating similar forms of trust and cultural connection elsewhere.

### Implications and future directions

Even with these limitations, several implications stand out. First, efforts to expand literacy opportunities in under-resourced communities must move beyond the distribution of books or materials to also consider the relational conditions that make literacy meaningful. Trusted messengers—whether librarians or Reading Captains—created contexts where families felt welcomed, affirmed, and motivated to participate. Second, the findings suggest that insider and outsider roles both have a place. Librarians brought professional scaffolding and institutional reach; Reading Captains brought cultural resonance and trust. Together they illustrate that equity is best served not by privileging one model over another, but by supporting multiple pathways that respond to community strengths.

For practice and policy, this means that scaling trusted messenger models requires more than replication. It calls for flexibility, allowing local communities to adapt the approach to their own histories, resources, and needs. It also calls for durable partnerships—among libraries, community organizations, schools, public health agencies, and funders—that can sustain these efforts over time. A public health lens further suggests the value of connecting trusted messengers to broader family-serving systems, including pediatric, maternal and child health, and community health initiatives, while preserving the local relationships that make the model credible. Finally, future research should examine the long-term effects of trusted messenger engagement: Do families sustain new reading habits? Do children carry forward gains in language, identity, and confidence? Can these models strengthen connection to other developmental or health-promoting resources? And how might trusted messengers help build bridges between informal community spaces and formal educational and health systems?

## Conclusion

At its heart, this study reminds us that early literacy does not unfold only in classrooms or through formal instruction. It emerges in the everyday places where families gather, and it is nurtured through trust and relationship-building. In laundromats across three cities, librarians and Reading Captains transformed ordinary routines into opportunities for reading, storytelling, and connection. Their practices differed, one rooted in professional structure, the other in relational familiarity—but both affirmed that children’s languages, cultures, and identities are assets to be celebrated. By centering trusted messengers, we see that literacy is not something delivered to families, but something co-constructed in the flow of daily life. For public health, the broader implication is that reducing disparities may require investing not only in formal services, but also in the community relationships and everyday settings that shape children’s opportunities to develop and thrive. These findings invite us to imagine more equitable developmental ecosystems, ones that recognize families as partners, communities as resources, and trust as a foundation for learning and health.

Perhaps the larger lesson is that expanding opportunity in communities does not always require building something new. It may begin by recognizing what communities already have: everyday places where families gather, people they know and trust, institutions willing to reach beyond their walls, and partners willing to invest in children. The challenge, then is to connect these assets in ways that make opportunities for learning more visible, more accessible, and more deeply rooted in community life.

## Data Availability

The datasets presented in this study can be found in online repositories. The names of the repository/repositories and accession number(s) can be found below: NYU Box.
